# Editorial: The role of nutritional interventions in optimizing exercise outcomes and recovery

**DOI:** 10.3389/fnut.2026.1986793

**Published:** 2026-09-18

**Authors:** Ellen Glickman

**Affiliations:** Exercise Science, Kent State University, Kent, OH, United States

**Keywords:** dietary interventions, exercise recovery, nutritional strategies, performance improvements, sports nutrition

The goal of this Frontiers in Nutrition—Nutrition and Exercise, Research Topic was to advance a new understanding of the application of how nutritional interventions can enhance exercise performance and the recovery process. The breadth of submissions reflect the wide array of the nutritional strategies currently being investigated, ranging from specific nutritional interventions such as caffeine, beetroot juice, creatine supplementation, and probiotics to broader dietary patterns such as the Mediterranean diet.

This Special Topics include 26 systematic reviews and empirical studies. The breadth of these submissions highlight the complexity of the relationship between nutrition and human performance. These contributions examined nutrition knowledge and its application across different populations, performance outcomes, intervention strategies, timing of nutritional intake, and environmental conditions. Collectively, these studies provide important insights into how nutritional strategies may be implemented before, during and after exercise to influence physiological responses, performance, and recovery.

By bringing together diverse approaches, populations, and experimental conditions, these contributions build upon and extend our understanding of the role of nutrition in human movement physiology. The study of food intake is itself a science, and understanding its influence on human physiology requires close examination beyond individual nutrients or isolated interventions. Nutritional interventions, dosages, timing, populations, exercise protocols, and environmental conditions can all contribute to different physiological and performance outcomes. Recognizing the complexity of methodology and perspective is essential for advancing a more integrated understanding of how nutrition shapes physiology, performance, and recovery across a diversity of contexts in which human movement occurs.

Moving forward, the diversity of topics highlights the importance of identifying when and for whom nutritional interventions are most effective. Greater attention to differences across populations, intervention characteristics, exercise demands, and environmental contexts may help understand nutrition, exercise, and recovery in different contexts. Research that integrates these factors across studies are increasingly important for the translation into findings for nutritional and exercise strategies that are both evidence based and responsive to situation and individual demands. Emerging technologies may eventually allow researchers and practitioners to continuously monitor physiological and biochemical markers—including hydration status, glucose concentrations, metabolic responses, and performance indicators—and integrate these data in real time. Such approaches could move the field beyond traditional assessments conducted before, during and after exercise toward more individualized and nutritional strategies. Ultimately, these advances may enable more personalized approaches to nutrition and exercise, supporting health, recovery and performance throughout the lifespan.

Therefore, the contributions to this specialized Research Topic demonstrate that nutrition and exercise physiology are deeply interconnected and that continued investigation across diverse populations, environments, interventions, and performance conditions is timely. By continuing to explore this complex interplay, we can expand our understanding of human performance while developing strategies that promote health, wellbeing, and the ability to perform across the lifespan.

